# IE1 of Autographa californica Multiple Nucleopolyhedrovirus Activates Low Levels of Late Gene Expression in the Absence of Virus RNA Polymerase

**DOI:** 10.1128/spectrum.03432-22

**Published:** 2022-12-13

**Authors:** Yong Qi, Shan-Shan Wang, Lu-Lin Li

**Affiliations:** a Hubei Key Laboratory of Genetic Regulation and Integrative Biology, College of Life Sciences, Central China Normal University, Wuhan, China; University of Florida

**Keywords:** AcMNPV, IE1, baculovirus, late gene expression

## Abstract

Early and late gene expressions of baculoviruses have been known to rely on host RNA polymerase II and a virus-encoded RNA polymerase, separately. In this study, we found that Autographa californica multiple nucleopolyhedrovirus (AcMNPV) recombinant bacmids with the individual RNA polymerase subunit genes deleted could support low levels of expression of a reporter gene under the control of the promoter of a typical late gene, *vp39*, in transfected Sf9 cells. Through multistep subcloning of a genomic library of the virus and transient expression assay analysis, *ie1* was identified to be the only viral gene that was responsible for activation of late gene expression in the absence of the viral RNA polymerase. Furthermore, IE1 was found to be capable of activating reporter gene expression from the promoters of additional late genes *polh*, *p6.9*, *odv-e18*, *odv-e25*, and *gp41*, independent of any additional viral factors. Deletion of *ie1* from the virus genome eliminated late gene expression. The IE1-activated late gene expression was enhanced by the viral *hr4b*. It was shown to be insensitive to inhibition of α-amanitin and did not appear to have stable transcription start sites. It is proposed that IE1 may serve to recruit newly synthesized viral RNA polymerase to viral DNA by activating low levels of pretranscription of the late genes to create an appropriate DNA conformation.

**IMPORTANCE** The late gene expression of baculovirus has been known to depend on the virus-encoded RNA polymerase, which consists of four subunits. The immediate-early gene *ie1* was found to be required for viral early gene expression, late gene expression, and DNA replication. How it functions in late gene expression remains unclear. In this study, we found that AcMNPV IE1 could activate low levels of gene expression from late gene promoters independently of any additional viral factors, with nonspecific transcription start sites. This new finding will shed light on the role of IE1 in the regulation of late gene expression and the understanding of the mechanism of late gene transcription initiation.

## INTRODUCTION

Like many other virus groups, baculovirus gene expression can be distinguished into early and late expression phases. Early expression and late expression can be further divided into immediate-early, delayed-early, late, and very late expression. Early studies showed that the early gene expression of baculoviruses is driven by host RNA polymerase II; late gene expression is initiated simultaneously, with or after, the onset of DNA replication and is dependent on a new RNA polymerases produced by early gene expression, which is resistant to α-amanitin, an RNA polymerase II inhibitor (1–4). Late gene transcription has been shown start in the core sequence A/G/TTAAG of late promoters, which is located upstream of the open reading frames (ORFs) (5, 6).

Autographa californica nucleopolyhedrovirus (AcMNPV) is the most-studied baculovirus. A comprehensive analysis of viral gene transcription products in AcMNPV-infected *Trichoplusia ni* cells in culture by 5'-random amplification of cDNA ends (RACE) and RNA sequencing showed that the viral mRNAs started from 216 sites upstream of 156 ORFs; 126 transcription start sites (TSSs) upstream of 101 ORFs contained the late promoter core sequence A/G/TTAAG; 92 TSSs did not contain late promoter sequences; there were both early and late transcription start sites upstream of 21 ORFs (7). It has been shown that most viral mRNA is transcribed from early genes at 6 h after infection; the peak level of early gene transcription products appear 6 to 12 h after infection; mRNAs transcribed from late gene promoters peak 12 to 18 h after infection or later (7). The mechanisms by which early and late gene transcription is initiated are not well understood.

AcMNPV RNA polymerase purified from infected insect cells is a protein complex composed of four subunits encoded by early expressed genes, *lef4*, *lef8*, *lef9*, and *p47* (8), which have homologs in all baculovirus genomes that have been sequenced. LEF8 and LEF9 contain sequences similar to the functional motifs of the catalytic active centers of the β and β' subunits of RNA polymerases in bacteria and eukaryotes (9–11). Bacterial and eukaryotic RNA polymerases typically consist of 5 to 15 subunits. β and β' and α and ω subunits form conserved enzyme active centers. LEF4 is an RNA 5'-capping enzyme (12). P47 is not related to RNA polymerase subunits from other organisms.

Apart from the genes encoding the RNA polymerase subunits, an additional 15 genes have been identified from the AcMNPV genome to be involved in late gene expression, including *ie1*, *ie2*, *lef1* to -*12*, *p47*, *dnapol*, *p143*, *p35*, and *pp3*1 (13, 14). In addition, *vlf1*, *lef2*, and *pk1* have been found to be involved in very late gene expression (15–17). Among these genes, *dnapol*, *p143*, *lef1*, *lef2*, and *lef3*, which encode a DNA polymerase, a helicase, a primase, a primase accessory factor, and a single-stranded DNA binding protein separately, as well as the immediate-early gene *ie1*, are essential genes for viral DNA replication, while *p35*, *lef7*, *lef11*, and *ie2* are stimulatory (18–20). The antiapoptotic factor gene *p35* likely promotes both viral DNA replication and late transcription by preserving the viability of cells. Immediate-early expressed gene *ie2* may affect late gene transcription by influencing DNA replication. *lef5*, *lef6*, *lef10*, *lef12*, and *pp31* are thought to be specifically involved in late gene transcription. LEF5 functions as a transcription initiation factor (21). LEF6 contains sequence homologous to the RNA binding domain of a retroviral mRNA export factor (22). LEF10 behaves as a prion (23). Prions in plants and yeasts also function as transcriptional regulators. The function of LEF12 is unknown. PP31 is a phosphoprotein that binds to single- and double-stranded DNA and is localized to the viral matrix of infected cells (24). Deletion of PP31 results in a significant decrease in the expression of several late genes of the virus (25).

AcMNPV *ie1* is the first immediate-early expression gene identified in baculoviruses. Its homologous genes are present in all α-baculoviruses and β-baculoviruses. IE1 protein was translated from two mRNAs, spliced and unspliced, which initiate transcription from different promoters (26). The unspliced mRNA encodes IE1 only. The spliced mRNA encodes IE1 and IE0 proteins. Compared with IE1, IE0 has an extra 54 amino acids at the N terminal. In 1986, AcMNPV *ie1* was first reported to be an activator for early gene expression. In a transient assay, the reporter gene controlled by AcMNPV *pp31* promoter could not be expressed in uninfected insect cell culture but was expressed in infected or transfected cell culture with a viral DNA fragment containing the *ie1* gene. The virus homologous repeat region (*hr*) sequence can significantly enhance the activation of early genes by IE1 (27, 28). More recent studies have reported that IE1 activates several early genes of the virus. IE1 is believed to play a central role in the early gene activation of baculoviruses (29). IE0 has also be shown to be able to activate early gene expression (30). Either IE1 or IE0 can support virus replication (31).

As IE1 is an early gene expression activator and DNA replication factor, it is speculated that IE1 may indirectly affect late gene expression by regulating other late gene expression factors and DNA replication. However, IE1, although initially expressed very early, maintains a high level of expression into the later stages of infection (32, 33). This distinguishing feature from other early expressed genes suggests that IE1 has some late regulatory function and may be directly involved in late gene expression.

In this study, analysis of AcMNPV RNA polymerase subunit deletion mutants created by AcMNPV RNA polymerase subunit deletion found that AcMNPV mutants with individual RNA polymerase subunit gene deletion could still drive low-level late gene expression in transfected cells. It was found that AcMNPV IE1 can activate gene expression from late promoters with nonspecific transcription start sites, independent of the viral RNA polymerase and other viral gene products in host cells.

## RESULTS

### Knockout of individual RNA polymerase subunit genes eliminated virus replication, but did not eliminate late gene expression.

**FIG 1:**
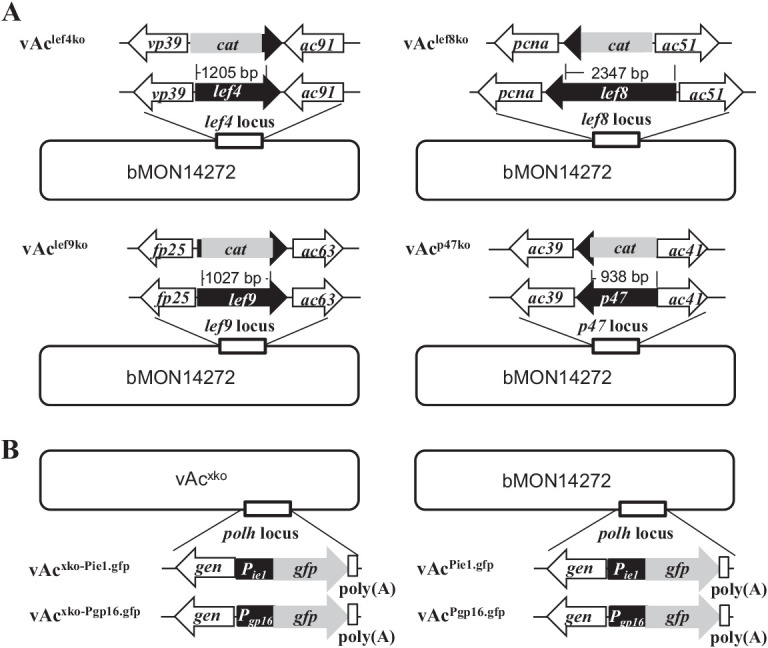

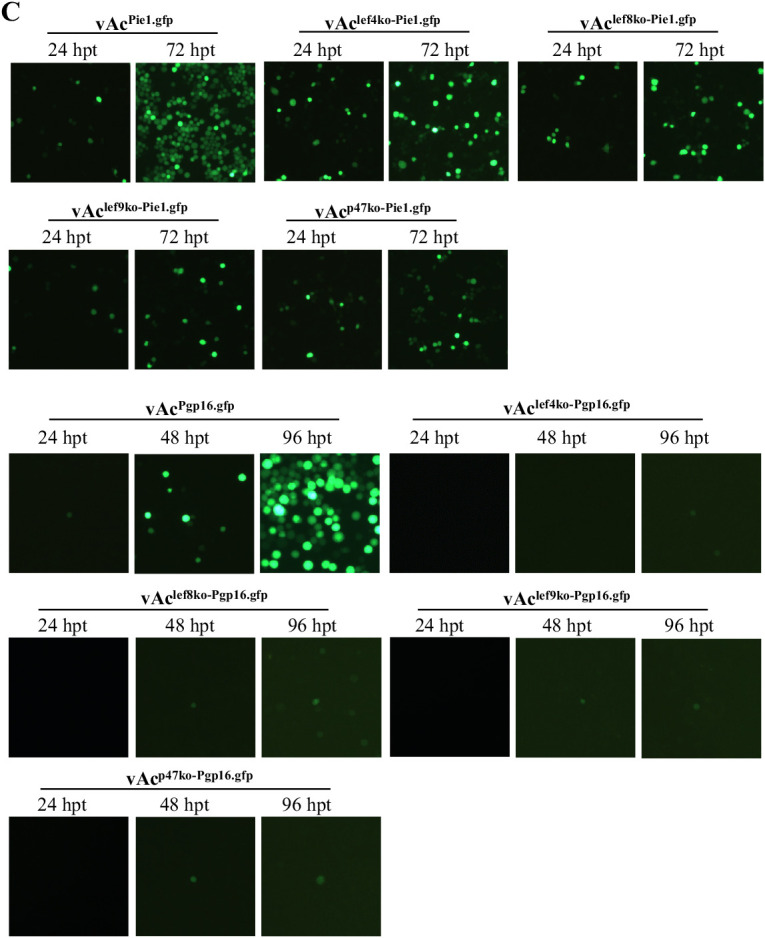

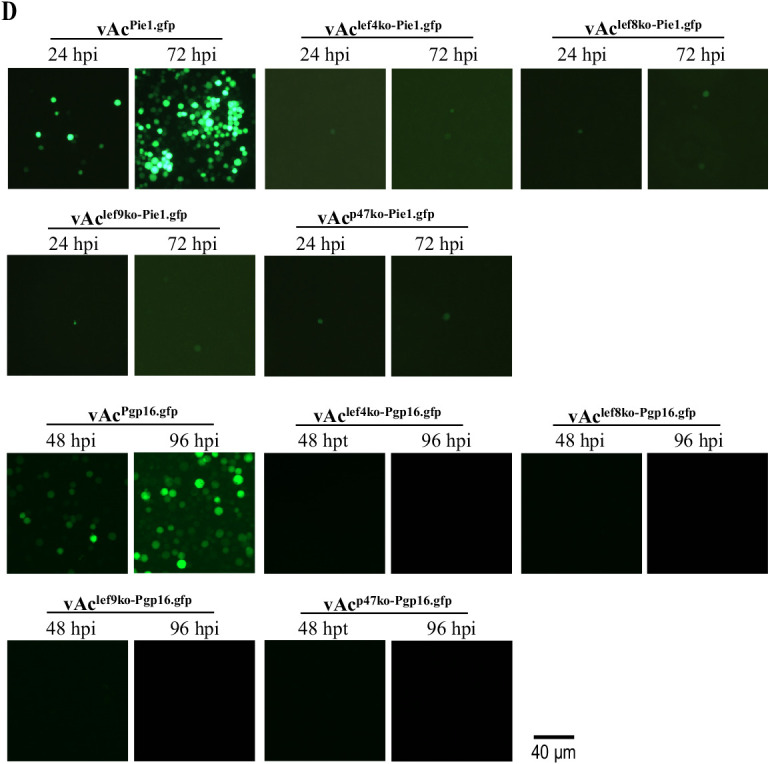
Construction and replication analysis of the AcMNPV recombinant bacmids with individual RNA polymerase subunit genes knocked out. (A) Schematic maps of the structures of the *lef4*, *lef8*, *lef9*, and *p47* locus in the wild-type AcMNPV bacmid bMON14272 and the recombinant bacmids vAc^lef4ko^, vAc^lef8ko^, vAc^lef9ko^, and vAc^p47ko^, in which most coding sequences of *lef4*, *lef8*, *lef9*, and *p47* were replaced with a copy of the chloramphenicol acetyltransferase gene (*cat*) gene. (B) Schematic maps of the structures of the *polh* locus in the AcMNPV recombinant bacmids vAc^lef4ko-Pie1.gfp^, vAc^lef4ko-Pgp16.gfp^, vAc^lef8ko-Pie1.gfp^, vAc^lef8ko-Pgp16.gfp^, vAc^lef9ko-Pie1.gfp^, vAc^lef9ko-Pgp16.gfp^, vAc^p47ko-Pie1.gfp^, vAc^p47ko-Pgp16.gfp^, vAc^Pie1.gfp^, and vAc^Pgp16.luc^. In these bacmids, a copy of *egfp* linked with a copy of the promoter of AcMNPV *ie1* (Pie1) or *gp16* (Pgp16) was inserted into the *polh* locus. “x” represents *lef4*, *lef8*, *lef9*, or *p47*. (C) Fluorescence microscopy images of Sf9 cells transfected with the individual bacmids containing *gfp* under the control of Pie1 or Pgp16. (D) Fluorescence microscopy images of Sf9 cells inoculated with the supernatant from the transfections described above, at designated time points posttransfection or infection.

In order to determine effects of individual subunits of viral RNA polymerase on virus replication, multiple mutants of AcMNPV with individual RNA polymerase subunit genes knocked out were constructed (Fig. 1A). In vAc^lef4ko^, vAc^lef8ko^, vAc^lef9ko^ and vAc^p47ko^, the first 1,205 nucleotides at the 5′-end of *lef4*, the first 2,347 nt at the 5′-end of *lef8*, 1,027 nt in the middle region of *lef9*, and the first 938 nt at the 5′-end of *p47* were knocked out and replaced by a copy of the *cat* gene, separately. A copy of *egfp* linked with the immediate-early gene *ie1* promoter P_ie1_ or the late gene *gp16* promoter P_gp16_ was inserted at the *polh* locus of the *lef* gene knockout mutants listed above to construct vAc^lef4ko-Pie1.gfp^, vAc^lef8ko-Pie1.gfp^, vAc^lef9ko-Pie1.gfp^, and vAc^p47ko-Pie1.gfp^, vAc^lef4ko-Pgp16.gfp^, vAc^lef8ko Pgp16.gfp^, vAc^lef9ko-Pgp16.gfp^, and vAc^p47ko- Pgp16.gfp^, respectively (Fig. 1B). The *egfp* gene served as a marker for gene expression and replication of the virus in transfected and infected host cells.

The effects of the deletions of the individual RNA polymerase subunit genes on virus replication were first examined by fluorescence microscopy of Sf9 cells transfected with the individual AcMNPV recombinants. As shown in Fig. 1C, fluorescence was observed in some cells in all the cultures transfected with the recombinant bacmids containing *egfp* under the control of the early promoter Pie1, as early as 24 h posttransfection (hpt). By 72 hpt, almost all cells in the culture transfected with vAc^Pie1.gfp^, which served as a wild-type control, were fluorescent, while less than half the cells in the cultures transfected with vAc^lef4ko-Pie1.gfp^, vAc^lef8ko-Pie1.gfp^, vAc^lef9ko-Pie1.gfp^, or vAc^p47ko-Pie1.gfp^ became fluorescent. These phenotypes suggested that the bacmids were transfected into some of the cells and immediate-early genes were expressed in these cells. In the cell cultures transfected with wild-type vAc^Pgp16.gfp^, weak fluorescence was first observed in a few cells at 24 hpt; almost all cells became brightly fluorescent by 96 hpt. In the cultures transfected with the bacmids lacking individual RNA polymerase subunit genes, weak fluorescence was also observed in a few cells at 48 hpt, but the number of the cells with fluorescence did not increase obviously and fluorescence observed in the cells was still weak at 96 hpt (Fig. 1D). These results were somewhat surprising, because *egfp* was under the control of a late promoter, Pgp16, in the bacmids. This implied that late gene expression occurred in the cells transfected with bacmids lacking individual RNA polymerase subunit genes.

To test if infectious budded virions were produced in the transfections, the supernatants collected from each transfection, at 120 hpt, were individually inoculated into fresh cultures of Sf9 cells. By 96 h postinoculation (hpi), almost all cells in the plates inoculated with supernatant of vAc^Pie1.gfp^ or vAc^Pgp16.gfp^ were filled with fluorescence (Fig. 1D), implying that both vAc^Pie1.gfp^ and vAc^Pgp16.gfp^ replicated and produced plenty of infectious progeny viruses in the cells transfected. In contrast, no fluorescence was observed by the end of the period in the cultures inoculated with the supernatant of the transfections with the individual recombinant bacmids lacking a RNA polymerase subunit gene, indicating that no infectious progeny virus was produced in transfection (Fig. 1D).

These results showed that missing any of the RNA polymerase-coding genes caused the virus to lose its ability to produce progeny viruses, but this did not seem to result in complete lack of expression of late genes.

### AcMNPV recombinants missing individual RNA polymerase subunit genes supported low levels of late gene expression.

**FIG 2:**
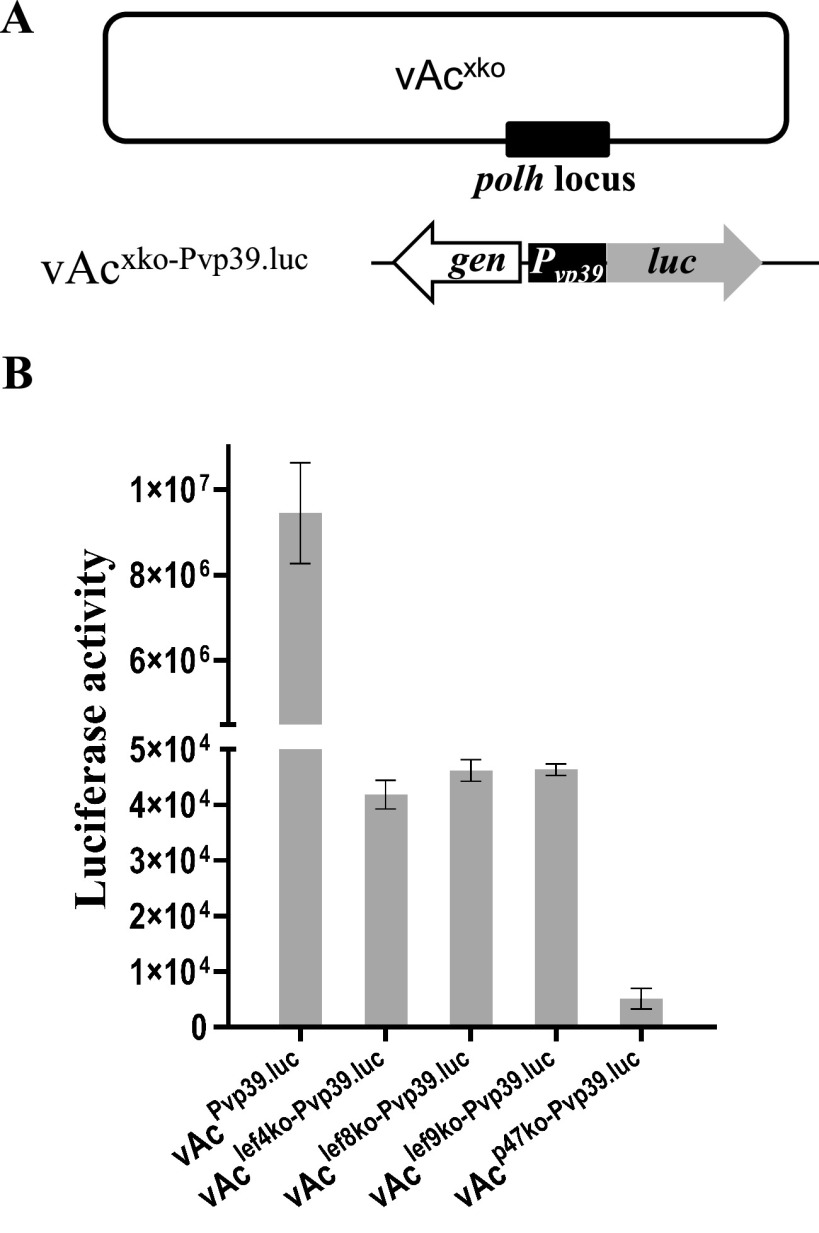
Effects of missing individual RNA polymerase subunit genes on late gene expression of AcMNPV. (A) Constructs of the late gene expression reporter bacmids missing individual RNA polymerase subunit genes, in which a copy of the luciferase gene linked with a Pvp39 was inserted at the *polh* locus. (B) Transient assay of late gene expression of the AcMNPV recombinant bacmids missing individual RNA polymerase subunit genes. The graphs show LUC activity (in relative light units) measured at 48 hpt in Sf9 cells transfected with the reporter bacmids vAc^lef4ko-Pvp39.luc^, vAc^lef8ko-Pvp39.luc^, vAc^lef9ko-Pvp39.luc^, vAc^p47ko-Pvp39.luc^, and vAc^Pvp39.luc^. Error bars represent the standard errors from three independent experiments.

To analyze quantitatively the effects of individual subunits of viral RNA polymerase on late gene expression, a copy of the *luc* gene linked with a copy of the promoter of the AcMNPV *vp39* gene, Pvp39, was inserted into the *polh* locus of vAc^lef4ko^, vAc^lef8ko^, vAc^lef9ko^, and vAc^p47ko^. The resultant late gene expression reporter bacmids vAc^lef4ko-Pvp39.luc^, vAc^lef8ko-Pvp39.luc^, vAc^lef9ko-Pvp39.luc^, and vAc^p47ko-Pvp39.luc^ (Fig. 2A) were used to transfect Sf9 cells seeded in a 96-well plate (1 μg/well), respectively. vAc^Pvp39.luc^ (34) were used as a wild-type control. Luciferase activity generated in each well was measured 48 hpt. As shown in Fig. 2B, the levels of luciferase activity expressed by vAc^lef4ko-Pvp39.luc^, vAc^lef8ko-Pvp39.luc^, vAc^lef9ko-Pvp39.luc^, and vAc^p47ko-Pvp39.luc^ and vAc^Pvp39.luc^ were 0.443%, 0.489%, 0.491%, and 0.054% relative to the level of that expressed by vAc^Pvp39.luc^. The LUC level expressed by vAc^p47ko-Pvp39.luc^ was significantly lower than levels expressed by the other RNA polymerase-deficient recombinants. Since *lef12* is located immediately upstream of *p47* in the opposite orientation, the promoter of *lef12* was deleted together with the ORF of *p47*. We are not sure whether this accounted for the reduction of the reporter gene expression.

These results proved that the AcMNPV recombinants lacking one of the four RNA polymerase subunit genes could support low levels of gene expression from the late promoter. Based on these results, it was difficult to determine whether the reporter gene expression controlled by the late promoter was catalyzed by an incomplete RNA polymerase lacking a single subunit or by another viral factor(s) or host enzyme.

### Identification of genomic elements responsible for activation of late gene promoters in the absence of viral RNA polymerase.

**FIG 3:**
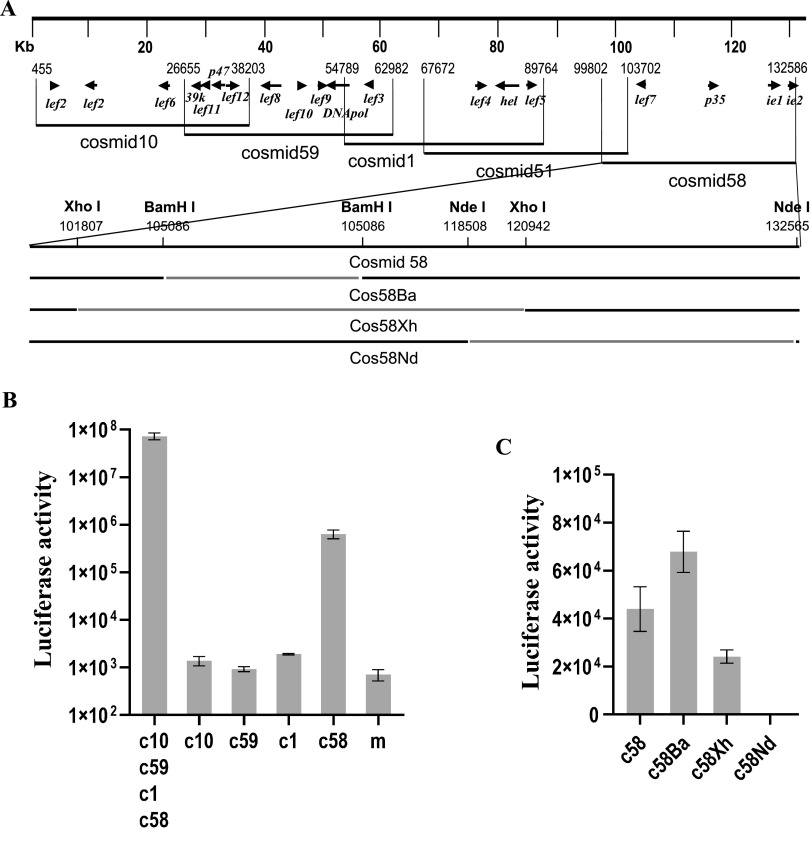
Screening of the genomic regions containing *cis*-elements associated with activation of late gene expression in the absence of viral RNA polymerase in a transient assay. (A) Maps of an AcMNPV cosmid library (13) and three subclones of cosmid 58. The restriction sites used for construction of the subclones and their locations on the genome are depicted. (B) LUC activity, measured at 48 hpt, in 10^6^ Sf9 cells cotransfected with pPacvp39.luc in combination with four cosmid clones or the individual cosmid clones. (C) LUC activity measured at 48 hpt in 10^5^ Sf9 cells cotransfected with pPacvp39.luc and the individual subclones of cosmid 58. The names of the cosmid clones are indicated below each column. c10, c59, c1, and c58 represent cosmid 10, cosmid 59, cosmid 1, and cosmid 59, respectively. Error bars represent the standard errors from three experiments. m, mock transfection.

Previously, a cosmid library was used in transient assays for identification of factors involved in late gene expression (13). The library consisted of five cosmid clones containing end-overlapping DNA fragments covering almost the whole genome sequence of AcMNPV (Fig. 3A). To identify the factor(s) associated with the activation of the reporter gene expression under the control of Pvp39, the individual cosmid clones were screened by transient assay in which the individual cosmid clones were transfected into Sf9 cells together with a reporter pPacvp39.luc that contained a copy of *luc* under the control of Pvp39 (34). As shown in Fig. 3B, a high level of luciferase activity was detected in the transfection with cosmid58 (c58), while only basic levels of LUC activity were detected in the other transfections, implying that the responsible genomic element(s) must be located in c58. Since no other genes encoding the RNA polymerase subunits are contained in c58, the reporter gene expression detected in this case should be irrelevant to the known viral RNA polymerase. c58 contains 37 ORFs (*ac116* to *ac152*) of the AcMNPV genome, including four late expression factor genes, *lef7* (*ac125*), *p35* (*ac135*), *ie1* (*ac147* and *ie0*), and *ie2* (*ac151*), and two *hr*s located downstream of *ac120* (*hr4c*) and *p35* (*hr5*), respectively (Fig. 3A).

In our next step, three subclones of c58 were constructed and used in transient assays, together with reporter plasmid pPacvp39.luc. As shown in Fig. 3C, the transfection with c58Ba missing *ac125* to *ac133* demonstrated a higher level of reporter gene expression than the one with c58, the level of reporter gene expression with c58Xh missing *ac119* to *ac138* was about 10% of that with c58, and no luciferase activity was detected in the cell culture transfected with c58Nd missing *ac136* to *ac152* including two *lef* genes, *ie1* and *ie2*.

**FIG 4:**
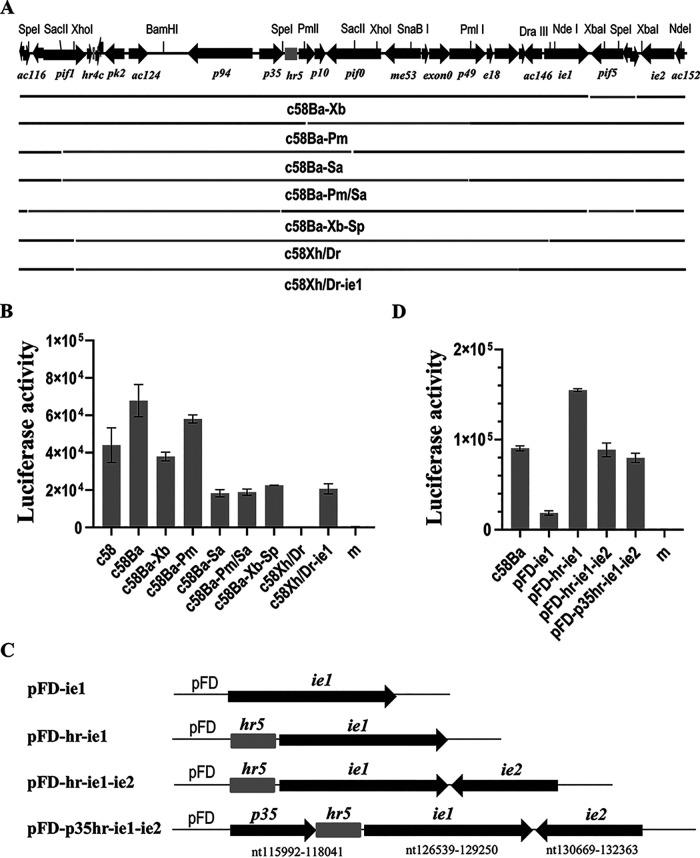
Identification of the genomic elements associated with activation of late gene expression in the absence of viral RNA polymerase in a transient assay. (A) Subclones of c58Ba. The restriction sites used for construction of the subclones and their locations on the genome are depicted. (B) LUC activity measured at 48 hpt in 10^5^ Sf9 cells cotransfected with pPacvp39.luc and the individual subclones of c58Ba. (C) Schematic maps of the transient expression plasmids containing *ie1*, or *ie1* and *hr5*, or *p35* and *ie2*. (D) LUC activity measured at 48 hpt in the cells cotransfected with pPacvp39.luc and the individual transient expression plasmids. The names of the cosmid clones or transient expression plasmids are indicated below each column. Error bars represent the standard errors from three experiments. m, mock transfection.

Since c58Ba activated high-level expression of the reporter gene, subcloning of c58Ba was done to screen out nonessential elements for activation of reporter gene expression (Fig. 4A). In transient assays, the level of reporter gene expression detected in the transfection with c58Ba-Pm missing *ac136* to *ac142* was higher than that with c58, although lower than that with c58Ba. The level in the transfection with c58Ba-Xb missing *ac148* to *ac151* was similar to the level with c58. The levels in transfections with c58Ba-Sa missing *ac119* to *ac138*, c58Ba-Pm/Sa missing *ac119* to *ac142*, and c58Ba-Xb-Sp missing *ac117* to *ac135* were 41.59% and 26.97%, 42.92% and 27.83%, and 51.19% and 33.19% of the levels with c58 and c58Ba, respectively (Fig. 4B). These results suggested that a genomic element(s) required for the reporter gene expression located in region between nt 124925 and 129250 containing *odv*-*e18*, *odv-ec27*, *ac145*, and *ie1* and/or the region between nt 130864 and n132586 containing *ie2* (most) and *ac152*. There likely were enhancing elements contained in the regions nt 101807 to 105149 and nt 113033 to 121005 regions containing *ac120*, *hr4c*, *ac121*, *ac122*, *pk2*, *ac124*, *p91*, *p35*, and *hr5*.

Subsequently, c58Xh was cut with DraIII to delete *odv*-*e18*, *odv-ec27*, *ac145*, and the 433-nt coding sequence at the 5′-end of *ie1* to make subclone c58Xh/Dr. Luciferase activity in the transfection with the resultant subclone c58Xh/Dr dropped to the background level. Then, a DNA fragment consisting of the 703-nt sequence upstream and the first 433 nt of the coding sequence of *ie1* was inserted back into c58Xh/Dr to repair *ie1*. A significant level of luciferase activity was detected in the cells transfected with the resultant cosmid, c58Xh/Dr-ie1, implying that *ie1* is essential for expression of the reporter gene. Next, a transient expression plasmid pFD-ie1 containing AcMNPV *ie1* with native promoter and transcription terminator sequence was constructed and used in a transient assay. We found that a significant level of luciferase activity was detected in the cells cotransfected with pFD-ie1 and Pvp39, but it was much lower than that with c58Xh/Dr-ie1 (Fig. 4B), implying that there must be an additional element(s) stimulating activation of reporter gene expression. Since either *hr4c* or *hr5* and *lef* genes *ie2* and *p35* are present in the subclones supporting high levels of reporter gene expression, *hr5* was inserted into pFD-ie1, alone or together with *p35* and/or *ie2*, to test their effects on activation of the reporter gene expression (Fig. 4C). As shown in Fig. 4D, a high level of luciferase activity was detected in the cells cotransfected with pPacvp39.luc and pFD-hr-ie1, which was much higher than those in the cells cotransfected with pPacvp39.luc and pFD-p35hr-ie1, pFD-hr-ie1-ie2, or c58Ba. *hr5* enhanced reporter gene expression about 8-fold, whereas *p35* and *ie2* did not appear to stimulate reporter expression activation.

### IE1 activated gene expression under the control of various late gene promoters.

**FIG 5:**
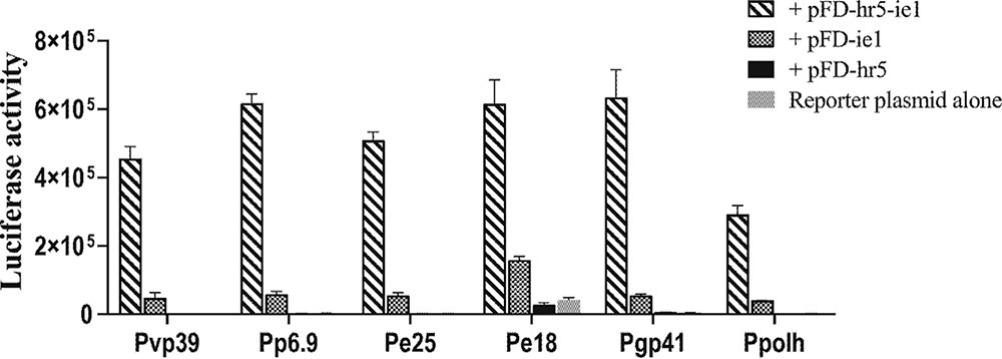
Comparative analysis of IE1-activated reporter gene expression from individual AcMNPV late promoters in transient assays. The graphs show LUC activity measured at 48 hpt in the cells cotransfected with individual reporter plasmids and a transient expression plasmid of *ie1* with or without *hr5* or with *hr5* alone. The late promoters contained in the reporter plasmids used in the experiment are indicated below each column group. Error bars represent the standard errors from three independent experiments.

Figure 5 shows results of transient assays on *ie1*-activated gene expression from various late gene promoters in addition to Pvp39. These included the promoters of the polyhedrin gene (Ppolh), the DNA-binding protein gene *p6.9* (Pp6.9), two major envelope protein genes, *odv-e25* and *odv-e18* (Pe25 and Pe18), and the tegument protein gene *gp41* (Pgp41). It can be seen that low levels of reporter gene expression were detected in all the cultures cotransfected with the individual reporter plasmids and pFD-ie1. The levels of reporter gene expression were elevated 3.9 to 11.6 times in the transfections with pFD-hr-ie1 relevant to the ones with pFD-ie1. No significant level of reporter gene expression was detected in the transfections with pFD-hr5 together with any one of the reporter plasmids. These results proved that IE1 activates expression of late genes universally. *hr5* likely stimulated reporter gene expression through enhancing *ie1* expression.

### IE1-activated gene expression from Pv39 was enhanced by an *hr*.

**FIG 6:**
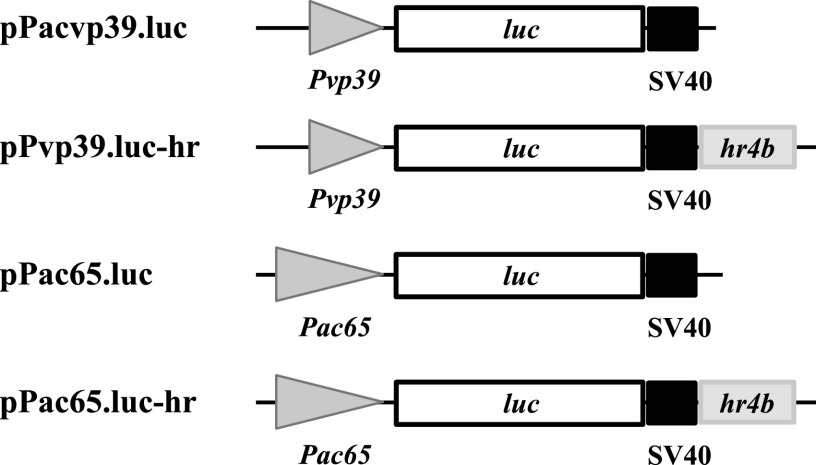
Schematic maps of late and early gene expression reporter plasmids with or without *hr*.

*hr* genes are known to enhance gene expression from early promoters (27, 28). To determine if the IE1-activated gene expression from late promoters could also be enhanced by an *hr*, we performed transient assays with reporter plasmids with or without an *hr*. In the late expression reporter plasmid pPvp39.luc-hr and the early expression reporter plasmid pPac65.luc-hr, a copy AcMNPV *hr4b* was inserted downstream of *luc* (Fig. 6). The results of transient assays showed that the LUC activity detected in the cells cotransfected with pPvp39.luc-hr and pFD-ie1 was 5.5 times the level in the transfection with pPacvp39.luc and pFD-ie1 and was about 1,000 times above the background level in the cells transfected with pPacvp39.luc alone (Table 1). *hr4b* enhanced the IE1-activated expression from Pvp39 4.5-fold. It also increased expression from the early promoter Pac65 about 70% in the presence of *ie1* (Table 1). The enhancement effect on Pvp39 was significantly higher than that on Pac65. The results also demonstrated that IE1 played a critical role in activation of gene expression from either the late promoter, Pvp39, or the early promoter, Pac65.

**TABLE 1:** Activation and enhancement of reporter gene expression^*a*^

Promoter	Transfected plasmid(s)	LUC activity/10^5^ cells	Fold enhancement
pPvp39	pPacvp39.luc	1,814 ± 9	0 (1)
	pPacvp39.lu + pFD-ie1	317,150 ± 4,244	1 (175.8)
	pPvp39.luc-hr + pFD-ie1	1,757,429 ± 169,146	5.5 (968.8)
pPac65	pPac65.luc	27,053 ± 1,583	0 (1)
	pPac65.luc + pFD-ie1	44,301,578 ± 2,231,306	1 (1,637.6)
	pPac65.luc-hr + pFD-ie1	74,538,054 ± 9,102,526	1.7 (2,755.3)

a The LUC activity values are averages ± SEM from three LUC assays. Fold enhancement values in parentheses represent the multiple of the level of reporter gene expression in the cells transfected with pPacvp39.luc or pPac65.luc separately.

### *ie1* is essential for activation of late gene expression.

**FIG 7:**
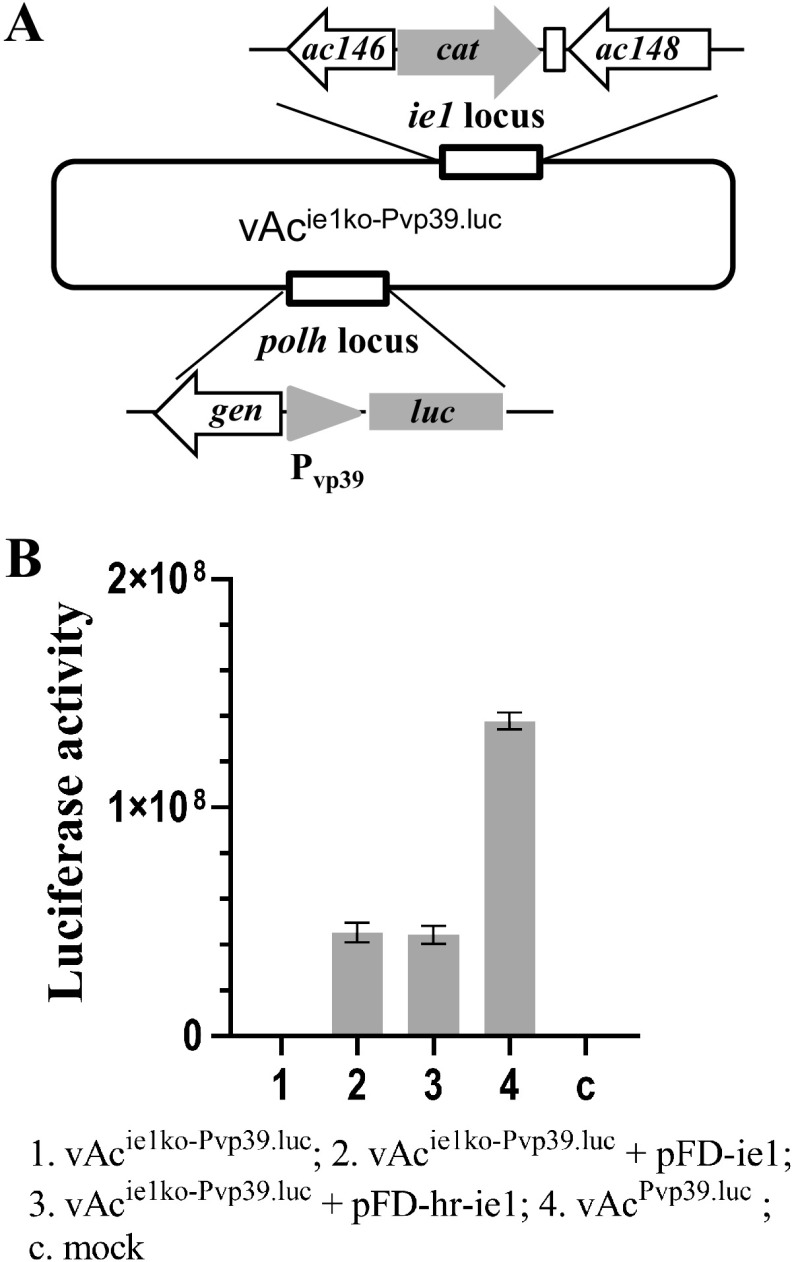
Requirement of *ie1* for late gene expression of AcMNPV. (A) Construction of the late gene expression reporter bacmid vAc^ie1ko-Pvp39.luc^, in which the first 1,678 nt of the *ie1* ORF were replaced with *cat* and a copy of the luciferase gene linked with a Pvp39 was inserted at the *polh* locus. (B) Results of a transient assay of late gene expression in Sf9 cells transfected with vAc^ie1ko-Pvp39.luc^ or cotransfected with vAc^ie1ko-Pvp39.luc^ and a transient expression plasmid of *ie1* with or without an *hr*. The graphs show LUC activities measured at 48 hpt. Error bars represent the standard errors from three independent experiments.

To verify the role *ie1* plays in activation of late gene expression during virus replication, an AcMNPV recombinant, vAc^ie1ko-Pvp39.luc^, with *ie1* replaced by a *cat* gene and with a copy of *luc* under the control of a late promoter P*_vp39_* inserted at the *polh* locus, was constructed (Fig. 7A) and used to transfect Sf9 cells. As a result, no luciferase activity was detected in the cells transfected with vAc^ie1ko-Pvp39.luc^, while significant levels of luciferase activity were detected in the cultures cotransfected with vAc^ie1ko-Pvp39.luc^ and pFD-ie1 or pFD-hr-ie1 at 48 hpt; these levels approximately one-third of the level detected in the cells transfected with vAc^Pvp39.luc^, which served as a positive control (Fig. 7B). The results proved that *ie1* is essential for activation of late gene expression of the virus during infection. The differences in reporter gene expression levels between the cultures cotransfected with the *ie1*-deficient mutant and pFD-ie1 or pFD-hr-ie1 and the culture transfected with vAc^Pvp39.luc^ should be attributed to the absence of IE0 in the former. However, in this case, the enhancement of IE1 expression by *hr5* apparently had no effect on late gene expression.

### IE1-activated gene expression controlled by a late promoter is resistant to an RNA polymerase II inhibitor.

**FIG 8:**
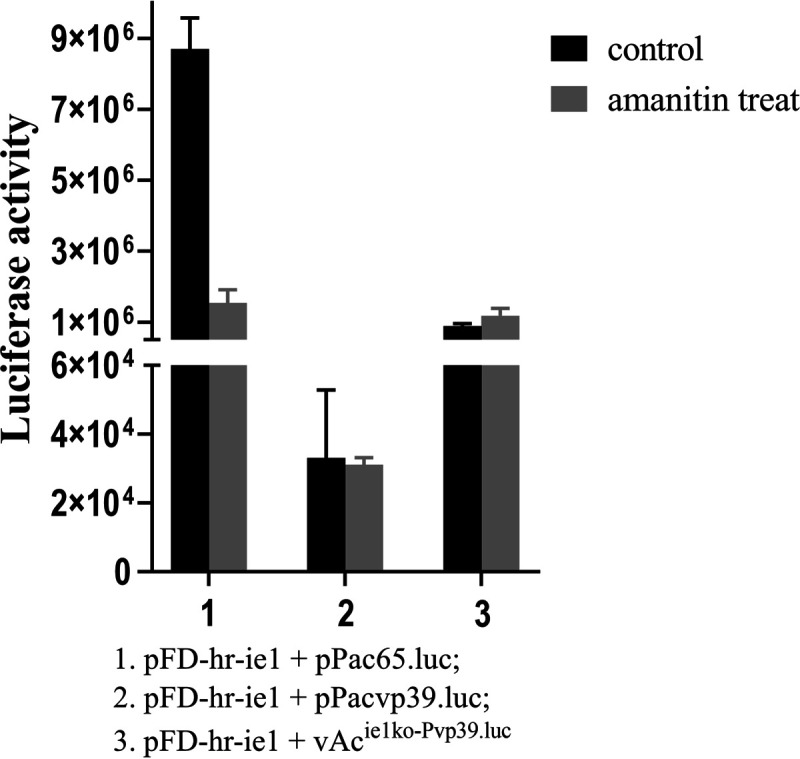
α-Amanitin inhibition assay of IE1-activated late gene transcription. Sf9 cells in culture were transfected with pFD-hr-ie1 at 12 h before addition of α-amanitin or water (control). Two hours after adding the inhibitor, the cells were subjected to transfection with the late expression reporter plasmid pPacvp39.luc or early expression reporter plasmid pPac65.luc. LUC activity in the cells was measured at 48 hpt. Error bars represent the standard errors from three independent experiments.

The experimental results above demonstrated that *ie1* could activate expression of reporter genes under the control of late gene promoters in the absence of a virus-encoded RNA polymerase, but it was not clear whether the RNA polymerase II of the host cells was involved in this event. To rule out the role the host RNA polymerase II might play in the process, α-amanitin was used to inhibit activity of cellular RNA polymerase II in transient assays. In the experiment, Sf9 cells in culture were transfected with pFD-hr-ie1 first and then incubated for 12 h, allowing expression of IE1 in the cells. Next, 0.5 μg/mL of α-amanitin was added, and the reporter plasmid mixed with transfection agent was introduced 2 h later. Luciferase activity in the cells was detected 48 h afterwards. As shown in Fig. 8, the level of luciferase activity in the cells transfected with early gene expression reporter plasmid pPac65.luc and treated with α-amanitin was reduced ~83% compared with the level in the cells without addition of α-amanitin. In contrast, luciferase activity in the cells transfected with late gene expression reporter plasmid pPacPvp39.luc and treated with α-amanitin was slightly lower than that in the cells with the same reporter plasmid and without addition of α-amanitin, but the difference was statistically insignificant. In the cultures transfected with reporter bacmid vAc^ie1ko-Pacvp39.luc^ that lacked *ie1* but contained a complete set of the RNA polymerase genes, luciferase activity was even elevated significantly by treatment with α-amanitin. These results showed that the early gene expression catalyzed by host RNA polymerase II was inhibited by α-amanitin while the late gene expression catalyzed by viral RNA polymerase was not inhibited, nor was the IE1-activated gene expression controlled by Pvp39 affected significantly.

### Transcription activated by *ie1* starts from nonspecific sites.

**FIG 9:**
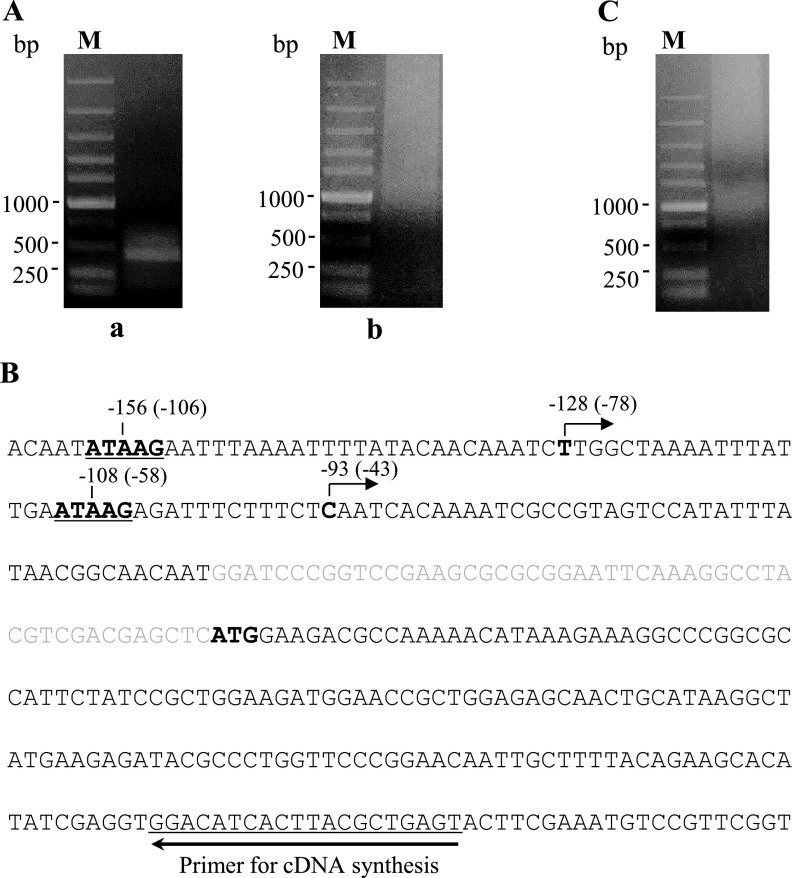
Identification of TSS of IE1-activated late gene expression in RACE assays. (A) Agarose gel electrophoresis of cDNAs of the RNAs transcribed from *luc* under the control of the Pvp39 in the cells cotransfected with pPacvp39.luc and c58Ba. In gel a, two separate cDNA fragments can be seen; in gel b, the cDNA electrophoresis showed a diffusion pattern. (B) Structures of the transcription cassette of the reporter gene *luc* under the control of Pvp39 in cells cotransfected with pPacvp39.luc and c58Ba. The TSSs were determined by sequencing the cDNA fragments shown in panel A, gel a. The TSSs are in bold, and transcription directions are indicated by arrows. The numbers above indicate their positions relative to the initiation ATG, and the numbers in parentheses indicate positions relative to the initiation ATG of the native *vp39* gene. The sequences of the primers used for synthesis of the first cDNA strand are underlined. (C) Agarose gel electrophoresis of cDNAs of the RNAs transcribed from *luc* under the control of Pvp39 in cells cotransfected with pPacvp39.luc and pFD-hr-ie1.

RACE analysis were performed to determine TSSs of gene expression under the control of late promoters in the absence of the viral RNA polymerase. Sf9 cells were cotransfected with pPacvp39.luc and c58Ba, and total RNAs of the cells were purified 48 hpt and used as templates for 5′-end amplification of *luc* transcript cDNAs. As shown in Fig. 9A (gel a), two cDNA fragments were detected. Sequencing results showed that the 5′ ends of these two DNA fragments corresponded to the −93C and −128T bases upstream of *luc* (−43 and −78 in the promoter of *vp39*), respectively, both locating between the two conservative late promoter core motif ATAAG (Fig. 9B). Notably, the TSS −43 is located in a CAAT motif that is frequently used as mRNA start site in insects and in baculovirus early genes (6, 35). However, consistent results were not obtained in the repeated experiments, in which, the electrophoretic pattern of the cDNA products was dispersive [Fig. 9A (gel b)]. A similar pattern, shown in Fig. 9C, was seen in the RACE analysis of the cells cotransfected with pPacvp39.luc and pFD-hr-ie1. We also performed RACE analysis of *odv-e18* transcripts purified from Sf9 cells transfected with cosmid 58, vAc^lef9ko-Pvp39.luc^, and vAc^Pvp39.luc^, respectively. The detected TSS of vAc^Pvp39.luc^ *odv-e18* was identical to that in previous reports (7) and localized to the TAAG motif, whereas the electrophoretic patterns of the cDNA products of the *odv-e18* transcripts purified from the cells transfected with cosmid 58 or vAc^lef9ko-Pvp39.luc^ were dispersive (data not shown).

## DISCUSSION

Baculovirus early gene expression depends on host RNA polymerase, and late gene expression depends on virus-encoded RNA polymerase. This has long been the consensus among scientists working with baculoviruses. In this study, we constructed several AcMNPV mutants missing one of the four genes encoding RNA polymerase subunits and proved that all four RNA polymerase subunit genes are essential for virus replication in transfection and infection experiments. Surprisingly, we found that AcMNPV recombinants lacking individual genes encoding RNA polymerase subunits and even a genomic clone of the virus not containing any RNA polymerase subunit gene could support low levels of gene expression from late gene promoters in transfected Sf 9 cells. Through screening, subcloning based on a viral genome library, and transient expression assays, *ie1* was identified to be the responsible genomic element for the late gene expression in the absence of the virus-encoded RNA polymerase. Furthermore, IE1 was shown to be able to activate gene expression from late promoters, independent of any other viral factors. In 1988, Guarino and Summers (27) reported low-level expression of the *ie1* antisense late promoter (likely the promoter of *ac148*) activated by IE1 in the presence of an AcMNPV enhancer *cis* linked to the late gene. Those findings demonstrated that IE1 is possibly involved in initiation of late gene transcription directly, corresponding to the persistent high level of IE1 expression throughout the viral replication cycle (7).

IE1 has been known to be an activator of early gene expression and to be essential for DNA replication and late gene expression (18, 19, 26). Previously, two independent functional domains located at the N terminal of IE1 were identified as responsible for activation of early gene transcription and DNA replication, differentially (36–38). The acidic activation domain for transcription was shown be exchangeable between IE1 homologs from different baculovirus species, the VP16 of herpes simplex virus, and transactivators from other viruses (39–41). The late genes of baculovirus may also be activated for transcription by the same activation domain of IE1. It is speculated that IE1 has the ability to interact with the RNA polymerase II-containing transcription complex and facilitate either the recruitment of other factors or to elevate the levels of transcription itself (42). In the absence of viral RNA polymerase, transcription of the late genes must be catalyzed by the RNA polymerase II or another enzyme of host cells. IE1 may play a role to recruit the RNA polymerase to the promoter regions of late genes. In this study, we performed RACE analysis on the transcripts of the reporter gene *luc* under the control of late promoter Pvp39 in cells transfected with cosmid 58 or pFD-hr-ie1 and of the late gene *odv-e18* in cells transfected with a *lef9*-deficient AcMNPV bacmid. We determined that two transcription start sites of the *luc* gene were localized outside of the conservative core sequence of the *vp39* promoter in the cells transfected with cosmid 58, but the result was not repeatable. In other cases, the amplified products were mixtures of indistinguishable sizes. These results suggested that the RNA polymerase involved binds the promoter with low affinity and initiates transcription from random sites. However, this low level of nonspecific transcription could serve to provide untangled DNA strand conformation that may be required for viral RNA polymerase to initiate transcription from specific sites of late promoters.

The RNA polymerase IIs in eukaryotes are sensitive to inhibition by α-amanitin, and the virus-encoded RNA polymerase of baculovirus has been shown to be resistant to the chemical (2). In this study, we found that IE1-activated gene expression from the late promoter Pvp39 was much more resistant to α-amanitin than the gene expression from the early promoter Pac65. This may reflect differences in properties between the RNA polymerase complexes binding the early and late promoters. In the cultures cotransfected with vAc^ie1ko-Pvp39.luc^ and pFD-hr-ie1, treatment with α-amanitin even stimulated reporter gene expression from Pvp39. This was reasonable, because late gene expression was catalyzed by the virus RNA polymerase in this case. Inhibition of redundant early gene expression would save resources for late gene expression.

In summary, we found that low levels of gene expression from late promoters occurred in the absence of the virus-encoded RNA polymerase in AcMNPV, and IE1 was the only required virus activator for it. Based on the existing data, we propose that IE1 serves to recruit host RNA polymerase onto the virus DNA. The RNA polymerase, recruited to viral DNA, travels along the DNA chain to initiate transcription from specific sites of early promoters. It also initiates transcription in late promoter regions randomly, with low efficiency, providing structural conformation for viral RNA polymerase to bind and initiate transcription from specific sites of late promoters. Alternatively, IE1 may be able to nonspecifically transcribe both early and late genes at low levels, recruiting host RNA polymerase and newly synthesized viral RNA polymerase to viral DNA by creating an appropriate DNA conformation.

The data from this study showed that *hr4b* significantly enhanced the IE1-activated expression of the reporter gene controlled by either an early or late promoter.

## MATERIALS AND METHODS

### Cell line, bacmids, cosmids, and plasmids.

The Sf9 cells (Invitrogen Life Technologies) were cultured at 27°C in Grace's medium containing 10% fetal bovine serum, penicillin (100 μg/mL), and streptomycin (100 μg/mL).

The AcMNPV bacmid bMON14272 derived from the AcMNPV strain E2 was maintained in DH10B cells as described previously (43).

Cosmid 10, cosmid 59, cosmid 1, and cosmid 58 are a group of cosmid clones containing end-overlapping DNA fragments that cover almost all of the genome of AcMNPV (13).

Subclones of cosmid 58 were constructed as follows: cosmid 58 was digested with BamHI, XhoI, and NdeI, individually, to delete nt 105149 to 113033, nt 101807 to 121005, and nt 118508 to 132565, respectively, of the AcMNPV DNA fragment and then recyclized by using T4 ligase. The recombinant cosmids were named c58Ba, c58Xh, and c58Nd, respectively. c58Ba was cut with XbaI, PmlI, and SacII, individually, or double-digested with PmlI and SacII to remove genome region nt 129250 to 130864 (*ac148* to *ac151*), nt 118308 to 124925 (*ac136* to *ac142*), nt 101357 to 120118 (*ac119* to *ac138*), or nt 101357 to 124925 (*ac119* to *ac142*) from c58Ba and then recyclized to make c58Ba-Xb, c58Ba-Sa, c58Ba-Pm, and c58Ba-pm/Sa, separately. c58Ba-Xb was cut with SpeI to remove viral genome sequence nt 100112 to 117348 (*ac117* to *ac135*) and then recyclized to make c58Ba-Xb-Sp. c58Xh was double-digested with XhoI and DraIII to remove nt 121006 to 127631 (me53, ac140, exon0, p49, odv-e18, odv-ec27, ac145, ac146, and 433 nt coding sequences at the 5′-end of *ie1*) and then recyclized to make c58Xh/Dr. A PCR fragment covering the 433-nt coding sequence of *ie1* and 703 nt upstream of the *ie1* ORF was inserted into c58Xh/Dr by homologous recombination using a pEASY-basic seamless cloning and assembly kit (Transgen). The resultant recombinant cosmid was named c58Xh/Dr-ie1.

The late expression reporter plasmids pPacvp39.luc, pPacpolh.luc, pPacp6.9.luc, pPace25.luc, pPace18.luc, pPacgp41.luc, and pPgp16.gfp were constructed as described previously (34, 44). These plasmids contain a copy of the luciferase gene (*luc*) or enhanced green fluorescence protein gene (*gfp*) under the control of the promoters of the designated AcMNPV late genes. The early expression reporter plasmid pPac65.luc was constructed by inserting *luc* under the control of the promoter of AcMNPV *dnapol* (nt 1 to ~600) between the SnaBI and BamHI sites of pFastBac 1 (Invitrogen). pPvp39.luc-hr and pPac65.luc-hr were constructed by inserting a copy of AcMNPV *hr4b* (nt 97456 to 97971) downstream of the simian virus 40 transcription terminator, separately, by homologous recombination using the pEASY-basic seamless cloning and assembly kit. pPie1.gfp was made by inserting *gfp* under the control of the promoter of AcMNPV *ie1*(nt −1 to ~750) between the SnaBI and PstI sites of pFastBac 1.

Transient expression plasmids used in the experiments were constructed as follows: PCR-amplified fragments nt 115992 to 118041 (contains *p35* and *hr5*), nt 117348 to 118041 (contains *hr5*), nt 126495 to 129250 (contains *ie1*), and nt 130669 to 132363 (contains *ie2*) of the AcMNPV genome, respectively. The initial ATG of *ac146* in the fragment from nt 126495 to 129250 was mutated to AGC. The fragments nt 115992 to 118041, nt 126495 to 129250, and nt 130669 to 132363 were ligated with pFastBac dual cut with EcoRI and XhoI by using the pEASY-basic seamless cloning and assembly kit to make pFD-p35hr-ie1-ie2. In the same way, the fragments nt 117348 to 118041, nt 126495 to 129250, and nt 130669 to 132363 were ligated with the linearized pFastBac dual to make pFD-hr-ie1-ie2; the fragments nt 117348 to 118041 and nt 126495 to 129250 were merged into pFastBac dual to construct pFD-hr-ie1; and the fragment nt 126495 to 129250 was merged into pFastBac dual to construct pFD-ie1.

### Knockout of individual RNA polymerase subunit genes from AcMNPV genome and reporter bacmid construction.

Knockout of individual RNA polymerase subunit genes from the AcMNPV genome was done with the method described by Datsenko and Wanner (45). Briefly, DNA fragments containing the chloramphenicol acetyltransferase gene (*cat*) cassette flanked by homologous arms of length from 40 bp to 539 bp corresponding to the sequences of AcMNPV genome nt 76558 to 78595 and nt 77801 to 77840 (for *lef4*), nt 40767 to 40806 and nt 43154 to 43193 (for *lef8*), nt 48718 to 49269 and nt 50296 to 50834 (for *lef9*), nt 32405 −32444 and nt 33383 – 33422 (for *p47*), nt 127163 – 127202 and nt 128871 – 128914 (for *ie1*), were synthesized and electrotransformed into arabinose-induced DH10B cells harboring bMON14272 and plasmid pKD46 encoding λ-Red recombinase, individually. White colonies growing on the plate with LB medium containing kanamycin, chloramphenicol, isopropyl-β-d-thiogalactopyranoside (IPTG), and 5-bromo-4-chloro-3-indolyl-β-d-galactopyranoside (X-Gal) were selected. Replacement of the individual target genes with the *cat* cassette was confirmed by PCR. The resultant recombinant bacmids were named vAc^lef4ko^, vAc^lef8ko^, vAc^lef9ko^, vAc^p47ko^, and vAc^ie1ko^, respectively.

A *gfp* or *luc* gene was inserted at the polyhedron gene (*polh*) locus of the individual gene knockout bacmids listed above to construct reporter bacmids, by transposition (43). Briefly, pFB-P_gp16_.gfp, pFB-P_ie1_.gfp, or pPacvp39.luc was transformed into Escherichia coli DH10B cells containing the individual gene knockout bacmids vAc^lef4ko^, vAc^lef8ko^, vAc^lef9ko^, vAc^p47ko^, vAc^ie1ko^, and pMON7124, separately. The recombinants with the reporter gene *gfp* or *luc* were selected on plates with LB medium containing chloramphenicol, kanamycin, gentamicin, IPTG, and X-gal. Insertion of the reporter genes was confirmed by PCR. The reporter bacmids generated were named as follows: vAc^lef4ko-Pgp16.gfp^, vAc^lef8ko-Pgp16.gfp^, vAc^lef9ko-Pgp16.gfp^, vAc^p47ko-Pgp16.gfp^, and vAc^ie1ko-Pgp16.gfp^; vAc^lef4ko-Pie1.gfp^, vAc^lef8ko-Pie1.gfp^, vAc^lef9ko-Pie1.gfp^, vAc^p47ko-Pie1.gfp^, and vAc^ie1ko- Pie1.gfp^; vAc^lef4ko-Pvp39.luc^, vAc^lef8ko-Pvp39.luc^, vAc^lef9ko-Pvp39.luc^, vAc^p47ko-Pvp39.luc^, and vAc^ie1ko-Pvp39.luc^.

All the transfer vectors above were sequenced to confirm construction. All of the bacmid constructs made by transposition were confirmed by PCR.

### Transfection and infection.

To perform transfection of Sf9 cells in culture, Sf9 cells were seeded in 35-mm plates (0.8 × 10^6^ cells/plate) or the wells of a 96-well plate (0.8 × 10^5^ cells/plate) and incubated at 27°C overnight. Then, a mixture of DNA and the FuGene HD transfection reagent (catalog number E2311, Promega) was added into the plates or wells, mixed, and incubated up to the designated time points. For transfection of the cells in a 35-mm plate, 0.5 μg of bacmid was mixed with 5 μL of the transfection agent and diluted into 100 μL with the growing medium. For transfection of the cells in a well of a 96-well plate with a bacmid or cosmid and/or a reporter plasmid, 100 ng of reporter plasmid and/or 2 μg of bacmid or cosmid was mixed with 1 μL of the transfection agent, diluted into 10 μL with medium, and let stand for 15 min. For the transfections, the subclones of cosmid 58, or the transient expression plasmids, a molecular number of subclone or plasmid matching that of 0.1 μg of cosmid 58 was added per well.

For infection, 1 × 10^6^ of Sf9 cells were seeded in a 35-mm plate and incubated at 27°C overnight. Then, the medium was removed, and 10 μL of infectious supernatant from the transfection with a wild-type bacmid or 500 μL of supernatant from a transfection with a recombinant bacmid with *ie1*, a RNA polymerase subunit gene was added into the plate, and incubation continued to the designated time.

### Transient assays of late gene expression.

Sf9 cells were seeded in wells of a 96-well tissue culture plate (1 × 10^5^ cells/well) and incubated at 27°C for 6 h, then transfected with individual reporter bacmids, or cotransfected with a reporter plasmid and a cosmid(s) or a plasmid(s). At designated time points posttransfection, the medium in the wells was removed, the cell layers were rinsed once with phosphate-buffered saline (PBS), and the cells were lysed by adding 100 μL of Glo lysis buffer (catalog number E605A, Promega) per well; the mixtures were incubated at room temperature for 10 min. Subsequently, 80 μL of the cell lysate from each well was transferred to the wells of the test plate and mixed with 10 μL of the Steady Glo luciferase assay substrate (catalog number E253A, Promega) that had been preadded in each well, and the mixtures were then incubated at 27°C for 5 min. Finally, the chemiluminescent reactions were measured using a CentroXS^3^ LB960 system (Berthold Technologies, Zug, Switzerland).

### Inhibition assays of RNA polymerase II activity with α-amanitin.

Sf9 cells was seeded in wells of a 96-well tissue culture plate (0.8 × 10^5^ cell/swell) and incubated at 27°C overnight. Then, the cultures were transfected with *pie1-hr* (25 ng/well). At 12 hpt, 0.5 μg/well of α-amanitin (to the final concentration of 5 microgram/mL) was added to each well, 2 h later the reporter plasmid (0.1 μg/well) or the reporter bacmid (2 microgram/well) was introduced by transfection; and after an additional 48 h of incubation, luciferase activities in the cell cultures were determined.

### RNA purification and RACE analysis of late gene transcripts.

Sf9 cells seeded in 35-mm plates (1 × 10^6^ cells/plate) were transfected with a cosmid or a bacmid or cotransfected with the reporter plasmid pPacvp39.luc and c58Ba. At 48h posttransfection, the medium in the culture was removed, and the cell layer was rinsed with PBS (prepared with diethyl pyrocarbonate [DEPC] in water) three times; then, 2 mL of TRIzol was added and the cells were suspended and lysed by gently blowing them up and down in a pipette. The cell lysate was transferred to an Eppendorf tube and incubated on ice for 10 min and then centrifuged at 11,400 rpm and 4°C for 10 min. The supernatant was mixed with 200 mL of chloroform with shaking for 15 s, incubated on ice for 15 min, and then centrifuged at 11,400 rpm and 4°C for 15 min. A 400-μL aliquot of the upper phase was taken and mixed with 500 μL of isopropyl alcohol, incubated at room temperature, and then centrifuged at 11,400 rpm for 10 min. The pellet was rinsed with 200 μL of 75% ethanol in DEPC water by centrifugation at 11,400 rpm for 5 min, air dried, and then dissolved in 20 μL of DEPC water. Two microliters of RNA sample was mixed with 1 μL of DNA digestion buffer, 1 μL of DNase I, and 6 μL of DEPC water and incubated at 37°C for 30 min. Then, 2 μL of 50 mM EDTA was added to inactivate DNase I by incubation at 65°C for 10 min.

First-strand cDNA synthesis and RACE analysis were done by using a SMARTer RACE 5′/3′ kit following the manufacturer’s instructions (TaKaRa). For first-strand cDNA synthesis, a mixture of 4.0 μL of 5× first-strand buffer, 0.5 μL dithiothreitol (100 mM), and 1.0 μL deoxynucleoside triphosphates (20 mM) was combined for the reaction buffer. Next, we mixed 1.0 μL of RNA, 1.0 μL of 5′-CDS primer A, and 9 μL of DEPC water, incubated the mixtures at 72°C for 3 min, cooled them down at 42°C for 2 min, and then spun them at 14,000 × *g* for 10 s, to obtain denatured RNAs. For the cDNA master mix, to the RNA sample we added 1 μL of SMART II A oligonucleotide and then 5.5 μL of 5′ RACE cDNA synthesis reaction buffer mix, 0.5 μL RNase inhibitor (40 U/μL), and 2.0 μL SMARTScribe reverse transcriptase (100 U). Finally, we combined 8 μL of the 5′ RACE cDNA synthesis master mix with the denatured RNA sample, incubated the mixture at 42°C for 90 min, and then heated it at 72°C for 10 min. We diluted the sample by adding 90 μL of Tricine-EDTA buffer to obtain 5′ RACE-ready cDNA. To perform RACE PCR, we mixed 25 μL of 2× SeqAmp buffer, 2.5 μL of 5′ RACE-ready cDNA, 5 μL of 10× universal primer A mixture, 1 μL of 5′ RACE primer, 1 μL of SeqAmp DNA polymerase, and 15.5 μL of PCR-grade water. The PCR conditions were as follows: first step, 94°C, 4 min; second step, 25 cycles of 94°C for 30 s, 68°C for 30 s, and 72°C for 3 min; third step, 72°C for 7 min.

### Data availability.

The data generated during the current study are available from the corresponding author upon request.
